# Comparison of clinical efficacy between robotic-laparoscopic excision and traditional laparoscopy for rectal cancer: A protocol for systematic review and meta-analysis

**DOI:** 10.1097/MD.0000000000020704

**Published:** 2020-07-02

**Authors:** Zhen Chen, Zhuo Li Zhu, Pingxi Wang, Fanwei Zeng

**Affiliations:** General Surgery Department, Dazhou Central Hospital, Tongchuan District, Dazhou, Sichuan Province, China.

**Keywords:** excision, laparoscopy, Leonardo's robot, minimally invasive surgery, rectal cancer

## Abstract

**Backgrounds:**

Laparoscopic surgery, robot-assisted surgery and open surgery are the most commonly consumed surgical techniques in daily living. Considering that in recent years, the situation of choosing laparoscopic surgery and robot-assisted surgery to treat rectal cancer in China is prosperous. Meanwhile, researches lacked in the comparison part between the 2, so we will systematically compare the clinical efficacy of robot-assisted resection and traditional laparoscopic resection for rectal cancer.

**Methods and analysis:**

We will search Clinical research literature published before January 2020 in PubMed, Embase, the Cochrane library, Science Network, Wan Fang database, Chinese national knowledge infrastructure, and Chinese biomedicine that evaluate the correlation of rectal cancer with Leonardo's robot and traditional laparoscopy, from inception to July 2019. Weighted mean difference and odds ratio were used to compare the efficacy of robot-assisted resection versus conventional laparoscopic resection for rectal cancer, and the main indicators are operation time, complication rate, conversion rate, blood loss, and length of stay.

**Results and conclusion:**

This study will systematically evaluate the clinical efficacy of robot-assisted resection and traditional laparoscopic resection for rectal cancer, thus providing evidence to the clinical application. The results will be published in a peer-reviewed journal.

**Ethics and dissemination:**

No ethical approval and participant consent are required, since this study data is based on published literature. The results of the study will be submitted to a peer-reviewed journal.

**PROSPERO registration number**: CRD42020172161

## 1 Introduction

Rectal cancer, the morbidity and mortality in our country are increasing year by year. Affected by diet and environment, the number of participants with rectal cancer has been high in China. Every year, a large number of participants with rectal cancer receive surgical treatment in the hospital. Moreover, the disease is also a major cause of death as well as a major public health problem in China, second only to lung cancer and gastric cancer participants, with an estimated 191,000 deaths in 2015.^[1]^ With the development and progress of the society, the demand for accurate treatment and minimally invasive surgery for tumors is on the increase.

Since the 1980s, minimally invasive techniques, represented by laparoscopy, have been developed and widely used due to their good clinical performance. Many data and records have proved that laparoscopic surgery is obviously superior to traditional open surgery in the effect, showing the advantages of less trauma and faster postoperative recovery. Many participants with rectal cancer also use laparoscopic surgery as their first choice for surgery. However, there are also some limitations, such as 2-dimensional plane imaging of the surgical field, and the increasing demands of counterintuitive reverse instrumentation. Last but not the least, the movement degree of freedom of the instrument is less, so it is difficult to complete fine separation, suture, anastomosis, and other operations.^[2]^

The Leonardo's robot surgical system is an advanced robotic platform that uses minimally invasive methods to perform complex surgery, radical prostatectomy, and a variety of gynecological procedures that currently make up the vast majority of robotic surgery. Since the robot system was first used for the treatment of rectal cancer in 2001, the robot system has been described as an effective tool for precise tissue separation and easier internal suture, and has been highly praised by surgeons.^[3]^

The robotic system allows the surgeon to operate without direct contact with the participant through 3D vision system (high definition, 10 times the magnification of visual stable camera) and operation control of action calibration system, Its flexible telescopic motion reduces physiological tremors, provides superior dexterity, and increases ergonomic comfort, perfectly addressing some of the drawbacks of traditional laparoscopic surgery.^[4]^

At present, more and more hospitals in China have begun to use the da Vinci surgical system for the treatment of rectal cancer. However, there is a major disadvantage of robot system in China, which is the high cost. Therefore, there are still a large number of hospitals in China that have not introduced robot system, and adopt traditional laparoscopic surgery instead. Consequently, there are a limited number of reports documenting the clinical outcomes of robotic versus laparoscopic surgery.

The purpose of our study was to conduct a systematic review and meta-analysis of robot-assisted and laparoscopic hepatectomy.

Since the rise and popularity of robotic systems from 2001 to 2010 was relatively short, the sample size of many previous meta-analyses was relatively small, which limited its statistical ability. So we took some studies published after 2014 as a reference,^[5–19]^ including more records and samples, which can significantly improve the sample size and statistical power of meta-analysis. Finally, it will be clear which approach is more beneficial to participants in terms of treatment effect and treatment cost.

## 2 Methods

### 2.1 Data sources and search strategy

This protocol is conducted according to the Preferred Reporting Items for Systematic Reviews and Meta-Analysis Protocol (PRISMA-P) statement guidelines. The search terms will include “Leonardo's robot”, “rectal cancer”, “laparoscopy”, “excision”, “minimally invasive surgery”. No language exclusions will be applied. The references of the identified studies will be manually searched

### 2.2 Study selection and inclusion criteria

We will include participants with any of the following conditions: rectal cancer (carcinoma of rectum or colorectal cancer) participants, we will include systematic reviews and meta-analyses that investigate the clinical efficacy between robotic-laparoscopic excision and traditional laparoscopy for rectal cancer. We will include any type of rectal cancer as experimental intervention (robotic-laparoscopic excision is used as adjunctive therapy while traditional laparoscopy as controls.).

### 2.3 Study design and outcomes

Clinical RCTs will be considered eligible for our study. Articles will be excluded from the current meta-analysis if they are duplicate articles, cohort studies, retrospective studies, case reports, letters, editorials, conference abstracts, or animal experimental studies. The primary outcome will be operation time, complication rate, conversion rate, blood loss, and length of stay.

### 2.4 Data extraction

Data extraction will include characteristics of systematic reviews and meta analyses (first author, publication year, the number of trials included, the number of participants in each meta-analysis, and methods used for pooled analysis), the interventions they received (name, dose, frequency, and the total duration of treatment), the monitoring for efficacy or adherence, and the measure of outcome (specifically defined as event or measure and time frame for the ascertainment of this outcome). For studies with more than 1 follow-up period, we will select the longest. In the event of missing data, we will attempt to contact the corresponding authors for details.

### 2.5 Assessment of methodologic quality

Two reviewers will independently appraise the methodological quality of the included studies using the Cochrane Collaboration tool for assessing the risk of bias. Each study will be reviewed and scored as having a high, low, or unclear risk of bias according to the following domains: selection bias (random sequence generation and allocation concealment), performance bias (blinding of participants and personnel), detection bias (blinding of outcome assessments), attrition bias (incomplete outcome data), reporting bias (selective reporting), and other bias (other sources of bias). The corresponding author would arbitrat any discrepancies between the findings of the reviewers.

## 3 Discussion

In this study, we evaluated the clinical efficacy of robot-assisted and conventional laparoscopic resection of rectal cancer. In general, we found that there was no significant difference in the safety of the 2 surgical methods. Except for the large number of low rectal tumors in the robot group, there was no statistically significant difference in surgical indicators among participants.^[5]^ However, the time of robot-assisted surgery is significantly higher than that of traditional laparoscopic surgery, the use of anesthetic painkillers is higher, and intestinal obstruction is significantly higher, which also increases the risk of complications of robot-assisted surgery. However, since the emergence of laparoscopic surgery is earlier than the manual assisted system, and surgeons have more time and experience to use it, it is not clear whether this finding is directly related to the early experience of surgeons and the fixed docking, start-up and disassembly time of robots. We plan to reassess the results in the future as we gain more experience and record more data on whether the time of robot-assisted surgery will be significantly reduced or even lower than that of traditional laparoscopic surgery.^[20,21]^

Conversion rate is one of the important parameters for the feasibility of this minimally invasive technique. Our meta-analysis showed the same results as the previous meta-analysis, that the conversion rate of robot-assisted surgery in rectal cancer resection was lower than that of traditional laparoscopic surgery. Our study shows that participants undergoing robot-assisted surgery generally spend more time in hospital than participants undergoing laparoscopic surgery.

There was no significant difference between the long-term assessment indicators, including the overall survival rate and the 2-year local recurrence rate. In none of our studies did we report details of participants who lost follow-up. Future studies should focus on long-term follow-up and evaluation of long-term outcomes of the da Vinci surgical system in participants with rectal cancer. Robot assisted surgery of rectal cancer participants need higher required upfront costs than traditional laparoscopic surgery participants. But the study of the evaluation results should be based on long-term treatment of participants with tumor and function. As a result, the overall cost efficiency has not yet been determined, and longer follow-up data is needed for further study. As a result, the overall cost efficiency has not yet been determined, and longer follow-up data is needed for further study.

## Author contributions

Zhen Chen and ZhuoLi Zhu conceived the idea for this study; Zhen Chen, ZhuoLi Zhu and Pingxi Wang designed the meta-analysis; Zhen Chen and Fanwei Zeng provided statistical advice and input; Zhen Chen drafted the protocol. Pingxi Wang and ZhuoLi Zhu reviewed the protocol and provided critical feedback. All authors approved the article in its final form.
